# AI literacy in healthcare organisations: implementing article 4 of the EU AI Act with M-SHALF

**DOI:** 10.1038/s44401-026-00104-0

**Published:** 2026-06-08

**Authors:** Sofia Palmieri, Jens Declerck, Sonia Priou, Nathan Lea, Dipak Kalra

**Affiliations:** The European Institute for Innovation through Health Data (i~HD), Ghent, Belgium

**Keywords:** Health policy, Information systems and information technology, Law

## Abstract

Article 4 of the EU AI Act mandates a ‘sufficient level of AI literacy’. We propose the Modular & Stratified Healthcare AI Literacy Framework (M-SHALF) to address role-specific knowledge needs across clinical, administrative, and technical domains. Framing literacy as governance infrastructure, we argue for a systemic approach to AI readiness that ensures ethical, compliant, and inclusive integration of AI in healthcare.

## Introduction

From triage desks to radiology suites, Artificial Intelligence (AI) is no longer a futuristic promise—it is actively reshaping the way healthcare organisations think, decide, and deliver care. Yet, as clinical and operational workflows become increasingly entangled with opaque algorithmic systems, healthcare organisations governance structures have not evolved at the same pace. This asymmetry between technological adoption and institutional readiness raises urgent questions about accountability, transparency, and trust.

The European Union’s Artificial Intelligence Act, adopted in 2024, represents a global milestone in efforts to regulate AI across sectors^1^. It seeks to promote the trustworthy development and use of artificial intelligence by requiring safe and reliable systems, while also ensuring that end users and affected individuals can engage with these tools confidently through access to clear information and relevant skills. Within it, Article 4 introduces a novel obligation: providers and deployers of AI systems must ensure a ‘sufficient level of AI literacy’ among individuals interacting with or affected by these technologies^2^. While the article signals a shift toward human-centred and anticipatory governance, its practical implementation remains deeply ambiguous— particularly in high-stakes environments like healthcare organisations. Terms such as ‘sufficient level’ or ‘to their best extent’ offer no standardised benchmarks or enforcement pathways, and the existing European Commission repository of AI literacy practices does not provide any example of healthcare organisations institutions implementing AI literacy practices^3^.

This contribution intervenes at the intersection of digital health and regulatory policy, asking: *What does AI literacy mean in the context of healthcare organisations governance? Who does it apply to, and how can it be operationalized in a way that is institutionally scalable, ethically robust, and legally sound?* Rather than treating literacy as a narrow compliance issue, we argue that it should be understood as part of the essential infrastructure for ethical and trustworthy AI integration in health systems. We propose a modular, stratified framework for AI literacy that accounts for the diverse roles and responsibilities within healthcare organisations, as well as their varying capacities for investment, potential barriers to implementation, and facilitators that support adoption - building toward a systemic vision of AI readiness that aligns with the goals of responsible and inclusive AI governance.

### AI in healthcare organisations: a complex deployment landscape

Artificial Intelligence is gradually making its way into healthcare, moving beyond a purely conceptual or future-facing role to selective implementation in healthcare organisations operations. While AI is beginning to influence clinical applications and back-office functions, its adoption remains uneven and often limited to pilot programs or specific use cases^1,4^. Many healthcare organisations have yet to fully integrate AI into their workflows, and where it is deployed, it is frequently done in an ad hoc manner without a centralised strategy or consistent understanding among staff. In clinical contexts, AI has become an integral part of diagnostic imaging, risk stratification, personalised medicine, and even robotic-assisted procedures^5–8^. Decision support systems are increasingly used to assist with differential diagnosis or treatment recommendations, raising questions about how clinicians engage with, trust, or challenge algorithmic outputs. These systems are not passive tools; they actively co-shape medical reasoning and workflow dynamics. As such, they alter the epistemic landscape of clinical care and demand a new kind of critical digital fluency among healthcare professionals^9–11^.

At the same time, AI is also being deployed in less visible but equally impactful domains. Administrative systems may use machine learning algorithms for workforce planning, patient triage, and bed allocation, often without the explicit awareness of the staff interacting with them^12–14^. Predictive analytics is also increasingly applied in operational decision-making—optimising discharge processes, reducing readmissions, or forecasting supply needs^15^. In many cases, employees are unaware that the systems guiding their day-to-day decisions are powered by AI systems. This opacity complicates accountability and reinforces the urgency of system-wide literacy.

When healthcare organisations don’t develop their tools in-house, they act as deployers, integrating external AI products into existing infrastructures. This makes the governance of AI especially challenging, as it requires both technical due diligence and institutional capacity to oversee, evaluate, and adapt these systems to local clinical and ethical standards^16^. Moreover, the rapid evolution of AI systems often outpaces organisational preparedness, leaving healthcare organisations to play catch-up in developing training, oversight mechanisms, and internal policies.

Crucially, the integration of AI systems in healthcare settings does not follow a top-down deployment model. In many cases, adoption is fragmented, driven by departmental interests, pilot initiatives, or vendor offerings. This decentralised implementation amplifies the variability of AI use across a single healthcare organisation, making a unified literacy strategy both more difficult and more necessary.

Understanding the heterogeneous nature of AI deployment in healthcare organisations is essential to framing any discussion on AI literacy. The range of roles, responsibilities, and degrees of exposure to AI among healthcare organisations staff means that literacy cannot be approached as a generic intervention. It must be tailored, context-sensitive, and stratified according to the diverse ways AI is embedded in practice. Without this nuance, efforts to comply with Article 4 of the EU AI Act risk becoming performative rather than transformative.

### Understanding Article 4 of the AI Act

Article 4 of the EU Artificial Intelligence Act introduces a legal obligation for all providers and deployers of AI systems to ensure a ‘sufficient level of AI literacy’ among their staff and other individuals involved in the operation and use of such systems. The provision states that measures should be taken ‘to their best extent’, and that literacy levels must be appropriate to the context of use, the individuals’ technical knowledge, experience, and training^2^. More broadly, Article 4 should be read in light of the AI Act’s overarching objectives—ensuring the safety of AI systems, strengthening accountability in their use, and protecting fundamental rights—which provide the normative framework within which the literacy obligation is situated.

On the surface, this appears to be a straightforward requirement: anyone working with or affected by AI systems must be educated about them. However, a closer reading reveals several interpretive challenges that complicate its practical implementation, especially in complex institutional settings like healthcare organisations.

First, the provision’s language is intentionally broad, even vague. Phrases such as ‘to their best extent’ or ‘sufficient level’ of literacy offer no standardised benchmark, leaving organisations uncertain about what exactly compliance entails. The distinction between ‘sufficient’ and ‘adequate’ (the latter used in Recital 91 of the Act) is left unexplained. Without clear metrics or assessment tools, organisations are left to define these standards themselves, raising concerns about inconsistency across sectors and institutions.

Second, the article specifies that literacy measures should apply to both ‘staff and other persons dealing with the operation and use of AI systems’, which significantly broadens the scope of responsibility. This wording implies that not only full-time employees but also contractors, temporary workers, and potentially even third-party service providers fall under the literacy requirement. The inclusion of ‘affected persons’ in Recital 20 further complicates the picture, suggesting that individuals impacted by AI decisions—such as patients—should be aware of and understand the role that AI is playing in care decisions that affect them, even if they have no direct interaction with the AI systems. Healthcare organisations need to ensure this literacy and awareness exists amongst its patients, in addition to its obligations towards it staff and contractors. While the binding force of recitals is limited, this framing implies that AI adopting organisations such as healthcare providers need to take a broad view of the persons, they need to ensure have adequate AI literacy.

Third, Article 4 situates AI literacy within a compliance system. Literacy is positioned as a tool to ensure that those deploying or using AI systems understand how to apply safeguards, interpret outputs, and oversee the technology effectively. However, this risk-based approach may narrow the ambition of AI literacy, framing it as a defensive strategy rather than a proactive educational and ethical undertaking.

To fully grasp the significance of Article 4, it must be understood as part of the broader regulatory architecture of the AI Act rather than in isolation. The AI Act aims to foster the trustworthy use of artificial intelligence, building safe AI systems, ensuring that end users and affected individuals feel confident in these tools by being well-informed about them and empowered with the skills needed to use them effectively. For instance, Article 13 on transparency obliges providers to design AI systems in a way that makes their functioning comprehensible to end-users, while Article 14 on human oversight requires that natural persons remain meaningfully involved in monitoring and intervening in AI systems’ operation. Together with Article 4, these provisions create an interdependent triad: literacy enables oversight and makes transparency actionable. Without literacy, transparency obligations risk becoming performative, and oversight duties unenforceable^17^. Articles such as Article 13 on transparency and Article 14 on human oversight apply specifically to high-risk AI systems under the AI Act's risk-based framework. A substantial portion of AI systems used in healthcare are likely to fall within this high-risk class because they are either intended for use in sensitive healthcare contexts deemed of high-risk *ex litteris* by the AI Act or qualify as regulate medical devices under EU harmonised legislations. However, the regulatory positions for AI used in healthcare remains flux. Proposed modifications concerning the interaction between the AI Act and the Medical Devices Regulation may alter the extent to which certain AI-enabled medical devices are automatically subjected to the AI Act high-risk requirements. In particular, the ongoing discussion surrounding the transition of the medical device regulation from Annex I Section A to Annex I Section B of the AI Act could narrow the direct applicability of some of the requirements for high-risk AI systems already regulated under the MDR. Should such modifications materialise, Article 4 would nevertheless continue to apply independently as a general obligation imposed on providers and deployers of AI systems but its interpretation would no longer be as directly reinforced through the operational logic of high-risk requirements. Yet, even if some MDR-regulated AI systems were no longer formally subjected to the high-risk requirements of the AI Act, these systems would operate in intrinsically high-risk clinical environments and should continue to receive heightened attention in the implementation of the AI literacy mandate. Against this backdrop, Article 4 emerges as a foundational governance mechanism within AI -enabled healthcare systems. For healthcare organisations, this means that human actors in AI-mediated systems must be equipped not only to operate technology, compliance but also to interpret, critique, and take responsibility for its actions considering particularly the risks that the specific use of that AI tool poses^18^. AI literacy functions as an enabling condition for the AI Act’s overarching objectives: it underpins accountability by ensuring actors can be held responsible for AI use, supports safety by equipping them to recognise and mitigate risks, and safeguards fundamental rights by enabling meaningful understanding and contestation of AI-driven decisions. In sum, while Article 4 establishes a clear legal obligation, it does not provide prescriptive answers to critical implementation questions: Who exactly does it apply to within a healthcare organisation? What depth of knowledge is required, and how can it be assessed? And most crucially, what kind of organisational structures are needed to translate this regulatory intention into action?

### Scope of Article 4 within healthcare organisations

One of the most immediate and complex challenges in applying Article 4 within a healthcare organisations setting is determining who exactly is covered by the literacy requirement. Unlike more vertically structured organisations, healthcare organisations are multi-layered institutions where AI systems are deployed across diverse domains, often with little coordination. Consequently, defining the relevant target groups for AI literacy is not merely a procedural task but rather a governance question that requires a detailed mapping of roles, systems, and workflows. At the same time, identifying ‘who’ falls under Article 4 cannot be separated from the broader logic of the AI Act. The obligations of transparency (Article 13) and human oversight (Article 14), as mentioned in the previous paragraph, presuppose that those engaging with AI systems possess at least a baseline level of literacy.

At the most basic level, the article clearly applies to staff who directly operate or interact with AI systems. This includes clinicians who use AI-assisted diagnostic tools, nurses who rely on AI-based triage or monitoring systems, and IT staff who configure, maintain, or update AI and data flow infrastructures. These individuals are not only users but often the first line of accountability for errors, malfunctions, or ethical dilemmas that AI systems may generate. Their literacy, therefore, must go beyond functional competence. In regulatory terms, these actors might embody the ‘human-in-the-loop’ envisioned in Article 14: they must be able to monitor, interpret, and, if necessary, override AI outputs. Without adequate literacy, their oversight role is reduced to a formality rather than a safeguard. A critical aspect of AI literacy for these professionals is the ability to recognise and respond to different types of errors—those that are immediately detectable during real-time execution or soon after, and those that only manifest months or years later when AI-driven care pathways prove suboptimal or even harmful. Understanding this distinction is essential, as it shapes how errors are identified, mitigated, and prevented, ensuring both immediate patient safety and long-term clinical efficacy. However, Article 4 also encompasses a broader category: ‘other persons dealing with the operation and use of AI systems’. In healthcare organisations, this could include administrative staff, legal and data protection officers involved in scheduling, billing, or admissions if those tasks are supported by AI systems^19^. Many of these individuals may not even be aware that they are engaging with AI, especially when AI is embedded into routine software or presented through opaque interfaces. For such groups, literacy obligations connect directly to Article 13 on transparency: awareness-based literacy ensures they can identify when AI is at work, understand the system’s outputs, and make sense of information disclosed by providers in compliance documentation. Without this capacity, transparency duties risk becoming performative rather than actionable.

Beyond day-to-day users, AI literacy should also be extended to those in administrative, procurement, and managerial roles who are responsible for selecting, purchasing, and overseeing the integration of AI systems into healthcare organisations infrastructures. These individuals play a pivotal role in shaping the institution’s technological landscape. Without a working knowledge of AI— its capabilities, limitations, and risks, as well as foundational concepts like data quality and data structures—procurement officers and administrative leads may lack the critical insight needed to evaluate the appropriateness and safety of proposed technologies. This knowledge is essential not only for identifying reliable systems but also for interpreting compliance documentation and anticipating potential downstream impacts. Here again, also Article 13 is relevant: procurement and managerial staff must be able to critically interpret provider disclosures about system performance, data governance, and risk management. Their literacy makes transparency enforceable in practice.

While discussions around enterprise-level responsibility for AI deployment remain, at present, largely a theoretical fascination among some U.S.-based legal scholars, they underscore a broader point: that institutional actors, not just technical operators, are implicated in the governance of AI^20^. Even in jurisdictions where corporate liability for AI harms is not clearly established, the ethical and strategic rationale for extending AI literacy to decision-makers is strong. Healthcare organisations that fail to ensure informed procurement and oversight risk not only technical failure, but also reputational and legal consequences in an increasingly regulated digital health landscape.

There is also a case to be made that healthcare organisations leadership and governance bodies—including department heads, compliance officers, legal teams, and board members—should be considered part of the target group under Article 4. These individuals may not engage with AI systems directly, but they are responsible for approving AI integration, overseeing risk management, and ensuring institutional compliance with the AI Act. In this capacity, their role links directly to Article 14: literacy equips them to exercise ‘human oversight’ at the strategic and governance level, ensuring that risks are identified and addressed before systems are deployed or even implemented in the healthcare organisation.

Furthermore, Article 4 raises questions about affected but non-operational groups, especially patients. While the article itself stops short of requiring healthcare organisations to educate patients, the inclusion of ‘affected persons’ in the Act’s recitals points toward a broader interpretation. Two distinct contexts must be considered when assessing patient literacy needs.

The first context involves a clinician using an AI system in diagnosis or treatment, with the patient as the recipient—whether beneficiary or, in some cases, unintended victim—of AI-informed decisions. In this scenario, the applicability of Article 4 does not extend to patients directly, and thus the obligation to build AI literacy among them does not fall within the scope of the healthcare institution’s legal responsibilities under this provision. The second context arises when patients themselves are the primary users of AI-enabled medical devices prescribed for home disease management—such as wearables, remote monitoring tools, or AI-powered insulin pumps. Here, the patient’s engagement is not passive but active, and their competence in using these systems safely and effectively becomes essential. While these patients fall outside the direct scope of Article 4, there is nonetheless an indirect institutional responsibility: healthcare organisations that prescribe or recommend such devices should take reasonable steps to ensure that patients reach a minimum level of competency. This may involve clear instructions, educational materials, or referral to support services. In this way, AI literacy becomes part of responsible care delivery, even if not explicitly mandated. While patient literacy may not be a legal requirement under Article 4, it emerges as an ethical extension of the same governance imperative. However, for the purposes of the framework developed below, patients are excluded, as the primary focus remains on internal healthcare organisations roles and responsibilities.

In practice, a healthcare organisation’s compliance with Article 4 will depend on how well it can identify its AI ecosystem and map literacy needs across its organisational strata. This requires a shift in thinking: from viewing AI literacy as a marginal training activity for a few specialised roles to understanding it as a core component of institutional governance. Every person who interacts with AI, makes decisions informed by it, or holds responsibility for its deployment must be equipped with the knowledge necessary to engage with it ethically and competently.

### AI literacy in practice

#### The modular and stratified healthcare AI literacy framework (M-SHALF)

Effective implementation of Article 4 of the EU AI Act within healthcare organisations settings requires a structured literacy strategy that reflects the complexity and heterogeneity of healthcare environments. Organisations vary considerably in size, resources, and baseline levels of digital literacy, which in turn shape their capacity to engage with AI literacy training. A modular and stratified framework offers a practical means of doing so—addressing the diverse knowledge needs, responsibilities, economic means, and risk exposures of healthcare organisations staff across clinical, technical, and administrative domains. In this way, the framework explicitly avoids a ‘one-size-fits-all’ approach and instead supports flexible, incremental adoption tailored to the institutional context. This level of structured detail, along with the delineation of layered roles and learning needs, goes beyond an illustrative example. It represents an advanced step toward formalising what multi-stakeholder AI literacy could look like in practice. In this light, we here display a proposal of the European institute of Innovation through Health Data (i~HD) Modular & Stratified Healthcare AI Literacy Framework (M-SHALF) (Supplementary Fig. 1) as a conceptual and operational model to guide implementation. The M-SHALF framework is organised across four progressive domains—Awareness, Application, Reflection, and Governance—which represent successive stages of maturity in AI literacy and organisational capability. These domains provide a scaffold for tailoring learning activities, responsibilities, and evaluation indicators according to institutional context and role-specific needs. Supplementary Figure 1 presents a high-resolution version of the M-SHALF conceptual structure; an accessible, zoomable format is provided in the Supplementary Table 1. Instead of attempting to redefine terms such as ‘*sufficient*’ and ‘*adequate*’ literacy—which remain deliberately broad within the regulation—M-SHALF offers a practical means for organisations to interpret and demonstrate them through role-specific competencies and continuous learning cycles, thereby translating regulatory intent into measurable literacy practices.

Moreover, the framework views AI literacy not as a static or uniform obligation but as a dynamic, role-dependent process that evolves in complexity and specificity. The levels of competence within the framework have been elaborated in more detail—though not shared here in full length—and are structured according to principles of AI literacy and Bloom’s taxonomy of educational objectives^21^ as already brought together by Ng et al.^22^. This approach differentiates between foundational knowledge (e.g. remembering and understanding AI concepts), applied skills (e.g. analysing system outputs, interpreting risks), and higher-order competences (e.g. evaluating algorithmic recommendations, creating governance strategies, or leading ethical decision-making processes). To support implementation and track meaningful engagement, the framework also introduces role-specific indicators aligned to each competency level. These indicators—ranging from knowledge-based quizzes and simulation exercises to audit reports—serve as operational benchmarks for evaluating both individual learning outcomes and institutional readiness. They also reflect regulatory alignment, assessing whether organisations are compliant with not only Article 4 (literacy), but also technical requirements related to CE-marking (the European conformity certification indicating that medical devices and software comply with EU safety, quality, and performance standards), conformity assessment, post-market monitoring, and risk management under Articles 8–15 of the AI ACT. While M-SHALF is anchored in the European regulatory framework, its modular and stratified architecture allows adaptation to other healthcare systems. The same principles of proportional implementation, professional role differentiation, and iterative governance can be mapped to different regulatory or accreditation environments—for example, those operating under the U.S. Food and Drug Administration’s SaMD guidance, Canada’s Medical Device Regulations, or national AI ethics frameworks. In such contexts, M-SHALF offers a transferable structure that can be aligned with local governance mechanisms while maintaining its educational and ethical foundations.

The M-SHALF framework is informed by an emerging literature review on AI literacy in healthcare. A targeted search was conducted in PubMed (complemented with Scopus) covering publications from 2018 to 2025, see Supplementary Table 1 (Part A—Methodological Details). We included conceptual papers, empirical studies, and educational initiatives addressing the competencies of healthcare professionals, administrative/governance staff, and IT professionals, and excluded purely technical contributions or non-healthcare contexts. This approach was not intended to be systematic but to identify the most directly relevant and recent contributions. The search identified approximately 20 publications, which primarily highlighted the need for AI education rather than specifying concrete competencies. To address these gaps, insights from expert round tables and competency-building initiatives at Ghent University and the Vlaamse AI Academie (VAIA) were incorporated. Evidence concerning administrative, legal, and governance professionals, as well as IT professionals, remains particularly sparse, necessitating the conceptual elaboration of competencies in these domains.

M-SHALF is indeed a framework proposal and not yet a validated protocol. We acknowledge that successful implementation will depend not only on defining competences but also on systematically addressing potential barriers and facilitators, which have been discussed in the broader literature on digital transformation and organisational change in healthcare. Reported barriers include resource constraints, limited training capacity, and institutional resistance to change, whereas facilitators typically encompass leadership endorsement, integration of literacy modules into existing professional development structures, and the adoption of phased, modular rollouts that enable organisations to progress at their own pace. These dynamics are also being actively explored in ongoing projects in which we are involved, aimed at operationalising AI tools in diverse healthcare settings and generating empirical evidence on organisational readiness and capacity-building strategies. In parallel, empirical validation of the M-SHALF framework is planned through expert and stakeholder roundtables, foresight exercises, and pilot implementations in healthcare organisations, which will allow systematic assessment of feasibility, acceptance, and impact. For a detailed roadmap illustrating phased implementation strategies, indicative timelines, and operational indicators across organisational contexts, see Supplementary Table 1 (B —Practical Implementation Roadmap).

Conceptually, the M-SHALF framework is broadly consistent with the Consolidated Framework for Implementation Research^23^, which offers a structured lens for understanding how new practices are adopted, adapted, and sustained in complex healthcare settings. While we do not presume to capture the full breadth of implementation science in this work, we considered it important to acknowledge this body of scholarship and to offer a preliminary reflection on how our framework aligns with its core concepts. In this regard, M-SHALF reflects CFIR’s five domains within its design logic, serving as a practical bridge between theoretical determinants of implementation and the operational realities of fostering AI literacy in healthcare organisations. The intervention characteristics are reflected in M-SHALF’s modular and stratified architecture, enabling proportional implementation according to an organisation’s size, digital maturity, and resource base. The outer setting is mirrored in the framework’s alignment with regulatory and policy drivers, including the EU AI Act and CE-marking requirements, which shape external incentives and compliance priorities. The inner setting is expressed through M-SHALF’s reflection and governance domains, which emphasise leadership engagement, interprofessional communication, and integration of literacy within organisational quality and risk-management processes. At the individual characteristics level, M-SHALF differentiates between AI literacy—a foundational understanding of AI concepts, implications, and responsibilities—and AI competency, the applied ability to critically use, evaluate, and oversee AI in practice^24^. Finally, the implementation process is embodied in the framework’s cyclical use of audits, training, and documentation to support continuous organisational learning and adaptive improvement. In this way, M-SHALF operationalises CFIR’s theoretical determinants, providing a practical structure for fostering sustainable AI literacy and readiness for trustworthy AI adoption in healthcare organisations.

For clarity, the framework distinguishes three key professional categories: (i) healthcare professionals (clinicians and allied health staff using AI in patient care); (ii) administrative, legal, and governance professionals (including managers, procurement officers, DPOs, and legal staff overseeing institutional compliance and ethical AI use); and (iii) IT and technical professionals responsible for AI system development, integration, and maintenance.

This paper has also highlighted the critical importance and ethical obligations for healthcare organisations to ensure that patients possess an adequate level of AI literacy when AI systems are being used in their care decisions. Without such literacy, patients may struggle to understand how AI tools contribute to their diagnosis or treatment recommendations, potentially leading to confusion, mistrust, or reduced engagement in shared decision-making. The M-SHALF framework does not presently provide a dedicated track addressing patient literacy and competence in relation to AI technologies. While healthcare organisations bear responsibility for transparency and communication, the comprehensive education of the wider public may be more effectively achieved through coordinated public education initiatives rather than being left solely to individual institutions. Such programmes could foster baseline understanding, empower patients to engage critically with AI-assisted care, and support equitable access to trustworthy health information. The authors are currently investigating this dimension of public education in more depth, and future publications will address strategies and models for enhancing patient and public AI literacy.

#### Structure

The foundation of the framework elaborated here is a core literacy module that introduces all healthcare organisations personnel to basic AI concepts, legal responsibilities, and the principles of trustworthy AI. This shared entry point is essential for establishing a common language and baseline of awareness across departments. It includes an overview of what AI is and is not, the risks and limitations associated with machine learning systems (such as bias or opacity), and the ethical and legal dimensions of AI deployment in healthcare. In some healthcare organisations settings this foundational module may need to begin one step earlier, by incorporating essential digital literacy training. AI literacy in such contexts—imagine a healthcare organisation transitioning from paper-based records to electronic health records—cannot be addressed in isolation; it must be embedded within a broader effort to build staff capacity in digital record-keeping, system interoperability, data entry standards, and cybersecurity. The goal is to ensure that staff are not only equipped to engage with AI systems but also able to navigate the digital infrastructures in which those systems operate.

Building on this foundation, the framework expands into three distinct learning tracks, each tailored to a key stakeholder group: healthcare professionals; administrative, DPOs, legal, and procurement personnel; and IT or technical staff. Each track increases in complexity and depth, aligning with the learner’s professional role, responsibilities, and interests. While the content is structured to meet the specific needs of each group, participants should be encouraged to engage with modules outside their primary track to gain a more holistic understanding of AI integration across the healthcare system.

For healthcare professionals, the next stage focuses on technical understanding of AI technologies and their application in clinical settings. This module aims to enhance critical appraisal skills, allowing clinicians to evaluate algorithmic recommendations, understand when and how to trust system outputs, and maintain clinical agency. A further module can then be offered on AI applications within specific medical domains—such as radiology, oncology, or pathology—enabling practitioners to interpret AI-assisted tools within their own disciplines. These sessions are ideally developed in collaboration with clinical societies and tailored to the evolving landscape of AI-enabled care. In practice, a growing number of professional associations and educational institutions have already begun incorporating AI-related competencies into continuing professional development programs, reflecting a broader movement toward preparing clinicians for responsible and informed AI integration in healthcare^10,24,25^. To ensure that literacy among healthcare professionals is both actionable and auditable, indicators include simulation-based assessments of AI-supported decision-making and documentation of override decisions in clinical records. Clinicians should also be familiar with whether the AI systems they use are CE-marked, what that certification entails, and what clinical validation data support their safe application. Advanced modules may require clinicians to evaluate post-market data, co-lead AI incident reviews, or contribute to ethical discussions on when AI should be deferred to or overruled. These indicators reflect a critical integration of literacy, responsibility, and regulatory awareness in direct patient care.

In parallel—and yet far less emphasised in the literature—administrative, legal, and oversight professionals, including those involved in procurement, compliance, and governance, also require a dedicated learning pathway. This track should focus on regulatory frameworks, institutional accountability, and ethical oversight. Foundational modules introduce the operational implications of the AI Act, such as obligations related to human oversight, documentation requirements, and post-market monitoring. As participants advance, the curriculum should include training in risk assessment, contract evaluation, and strategic decision-making concerning the adoption and governance of AI systems within healthcare organisations infrastructures. Importantly, these professionals must also gain a clear understanding of AI liability, patient rights, and the legal implications of algorithm-driven decision-making in clinical environments—ensuring institutional readiness not only for compliance but also for the protection of fundamental rights in AI-enabled care. In this track, indicators include the use of procurement checklists, understanding of AI-specific clauses in contracts (such as for liability, post-market surveillance, and human oversight), and documented reviews of the EU Declaration of Conformity and technical documentation required under Articles 10–11. Legal and governance professionals should also be trained to ensure that CE-marked systems meet their risk classification, that DPIAs are adapted to account for AI’s dynamic risk profiles, and that—when deemed appropriate—informed consent forms clearly explain AI involvement. These actions demonstrate that AI literacy is fully integrated with institutional due diligence and regulatory alignment, rather than treated as a one-time or peripheral training activity.

A third track is designed for IT and technical teams, who manage the integration, configuration, and monitoring of AI systems. Their pathway begins with understanding how AI systems function technically and how they interface with existing health information systems. It then advances to address data quality, bias mitigation, auditability, and technical documentation. At the highest level, this track equips staff with the competencies required to implement secure and explainable AI solutions in high-stakes clinical environments. In this context, indicators include the presence of system-level monitoring dashboards, documentation of model performance audits, and the active logging of system behaviour for post-market surveillance. Technical staff should also be equipped to review and manage the lifecycle of CE-marked AI systems, including updates, retraining, and verification of real-world performance across diverse populations. These indicators provide evidence that IT teams are not simply maintaining systems but actively governing them in line with both ethical expectations and regulatory mandates.

## Conclusion

The requirement for AI literacy under Article 4 of the EU AI Act offers a timely opportunity to strengthen institutional preparedness in healthcare organisations—but only if interpreted as more than a bureaucratic checkbox. While the regulatory mandate is legally binding, its true potential lies in fostering deeper ethical engagement, interpretive competence, and shared accountability across the healthcare organisations ecosystem. In this sense, AI literacy is not a peripheral training issue—it is a structural condition for meaningful human oversight, algorithmic transparency, and responsible deployment.

Yet the current state of readiness is uneven. Most healthcare organisations lack the governance infrastructure, curricular frameworks, and implementation tools needed to meet the regulatory threshold, let alone to advance toward best practice. Few have mapped their internal AI ecosystems or assessed who among their staff is directly or indirectly affected. Without a role-sensitive, technically grounded, and ethically informed approach to literacy, compliance efforts risk becoming symbolic rather than substantive.

To avoid this outcome, healthcare organisations should adopt a modular and stratified literacy model embedded in institutional governance. But individual institutions cannot act alone. There is an urgent need for coordinated support at the regional, national, and EU levels—including shared toolkits, open-access training content, and the inclusion of healthcare organisations in public repositories of AI literacy practices. Policymakers and research institutions must also recognise healthcare organisations as critical sites of AI governance—not just as users of AI, but as co-creators of responsible innovation.

## Supplementary information

Supplementary information

## References

[1] European Commission, Directorate General for Health and Food Safety, PwC EEIG & Open Evidence. *Study on the Deployment of AI in Healthcare: Final Report*. Publications Office; 2025.

[2] Fernandes E, Holmes W. General provisions, Article 4 AI literacy. In: *The EU Artificial Intelligence (AI) Act: A Commentary* (Kluwer Law International, 2024).

[3] Living Repository of AI Literacy Practices. 2025. Available at: https://digital-strategy.ec.europa.eu/en/library/living-repository-foster-learning-and-exchange-ai-literacy.

[4] Poon, E. G., Lemak, C. H., Rojas, J. C., Guptill, J. & Classen, D. Adoption of artificial intelligence in healthcare: survey of health system priorities, successes, and challenges. *J. Am. Med. Inform. Assoc.* **32**, 1093–1100 (2025). 40323320 10.1093/jamia/ocaf065 PMC12202002

[5] Iftikhar, M., Saqib, M., Zareen, M. & Mumtaz, H. Artificial intelligence: revolutionizing robotic surgery: review. *Ann. Med. Surg.* **86**, 5401–5409 (2024). 10.1097/MS9.0000000000002426 PMC11374272 39238994

[6] Adegbesan, A. et al. From scalpels to algorithms: the risk of dependence on artificial intelligence in surgery. *J. Med. Surg. Public Health.* **3**, 100140 (2024).

[7] Bhandari, A. Revolutionizing radiology with artificial intelligence. *Cureus* **16**, e72646 (2024). 39474591 10.7759/cureus.72646 PMC11521355

[8] Awasthi, S. et al. Identification and risk stratification of coronary disease by artificial intelligence-enabled ECG. *eClinicalMedicine* **65**, 102259 (2023). 38106563 10.1016/j.eclinm.2023.102259 PMC10725070

[9] Epistemo-ethical constraints on AI-human decision making for diagnostic purposes. *Ethics Inf. Technol*. **24**, 1–16 Available at: https://link.springer.com/article/10.1007/s10676-022-09629-y (2023).

[10] Schuitmaker, L., Drogt, J., Benders, M. & Jongsma, K. Physicians’ required competencies in AI-assisted clinical settings: a systematic review. *Br. Med. Bull.* **153**, ldae025 (2025). 39821209 10.1093/bmb/ldae025 PMC11738171

[11] Schubert, T., Oosterlinck, T., Stevens, R. D., Maxwell, P. H. & van der Schaar, M. AI education for clinicians. *eClinicalMedicine* **79**, 102968 (2024). 39720600 10.1016/j.eclinm.2024.102968 PMC11667627

[12] Artificial Intelligence for Patient Flow. Canadian Agency for Drugs and Technologies in Health; 2024. 38985917

[13] Da’Costa, A. et al. AI-driven triage in emergency departments: a review of benefits, challenges, and future directions. *Int. J. Med. Inform.* **197**, 105838 (2025). 39965433 10.1016/j.ijmedinf.2025.105838

[14] Tyler, S. et al. Use of artificial intelligence in triage in hospital emergency departments: a scoping review. *Cureus* **16**, e72646 (2024). 38854295 10.7759/cureus.59906 PMC11158416

[15] Malik, A. & Solaiman, B. AI in hospital administration and management: ethical and legal implications. In: *Research Handbook on Health, AI and the Law* (Edward Elgar Publishing, 2024). 40245223

[16] A guide to managing interconnected AI systems. *Harvard Business Review*. 2024 Dec. Available at: https://hbr.org/2024/12/a-guide-to-managing-interconnected-ai-systems.

[17] Panezi, A. Requirements of high-risk AI systems: AI Act. Article 14. Human oversight. *SSRN*. 10.2139/ssrn.5131229.

[18] Schneider-Kamp, A. & Godono, A. The AI-extended professional self: user-centric AI integration into professional practice with exemplars from healthcare. *AI Soc.* **40**, 1–14 (2025).

[19] AI literacy requirements under the EU AI Act: what healthcare leaders need to know. *HIMSS*. 2025. Available at: https://www.himss.org/news/ai-literacy-requirements-under-eu-ai-act-what-healthcare-leaders-need-know.

[20] Price II WN, Cohen IG. Locating liability for medical AI. *SSRN*. 2023. Available at: 10.2139/ssrn.4517740.

[21] Bloom, B. S., Engelhart, M. D., Furst, E. J., Hill, W. H. & Krathwohl, D. R. *Taxonomy of Educational Objectives: The Classification of Educational Goals*. Handbook I: Cognitive Domain. Longmans, Green, 1956.

[22] Ng, D. T. K., Leung, J. K., Chu, S. K. & Qiao, M. S. Conceptualizing AI literacy: an exploratory review. *Comput. Educ. Artif. Intell.* **2**, 100041 (2021).

[23] Damschroder, L. J., Reardon, C. M., Opra Widerquist, M. A. & Lowery, J. C. The updated consolidated framework for implementation research based on user feedback. *Implement. Sci.* **17**, 75 (2022). 36309746 10.1186/s13012-022-01245-0 PMC9617234

[24] Cookson, C. EU project launched to prepare health workers for a digital future. *Financial Times*. 2025.

[25] Liaw, W., Kueper, J. K., Lin, S., Bazemore, A. & Kakadiaris, I. Competencies for the use of artificial intelligence in primary care. *Ann. Fam. Med.* **20**, 559–563 (2022). 36443071 10.1370/afm.2887 PMC9705044

