# Supplementary material for: AI literacy in healthcare organisations: implementing article 4 of the EU AI Act with M-SHALF
Source: Npj Health Syst. 2026 Jun 8;3:42. doi: 10.1038/s44401-026-00104-0 (PMC13354241; doi:10.1038/s44401-026-00104-0)
Supplement: Supplementary file 1 — Supplementary information [file 44401_2026_104_MOESM1_ESM.pdf]

Supplementary Information A. Methodological Details

A.1 Literature Search Strategy

The development of the Medical AI Shared Literacy Framework (M-SHALF) was informed by an integrative literature review conducted between January 2018 and May 2025. The purpose of the review was to identify existing conceptualizations of AI literacy and competency within healthcare and related educational contexts.

Databases and Search Terms

The primary search was conducted in PubMed, using the following Boolean expression:

("Health Services Administration"[Mesh] OR "Hospital Administration"[Mesh]) AND ("artificial intelligence literacy" OR "AI literacy")

Equivalent terms for "healthcare professionals" were also tested using relevant MeSH expressions. Supplementary searches were conducted in Scopus and Web of Science to ensure comprehensive coverage.

Inclusion and Exclusion Criteria

Inclusion Criteria	Exclusion Criteria
Publications defining the essence of AI literacy or identifying new competencies required by healthcare professionals (HCPs).	Articles discussing the need for AI education without specifying competencies or content.
Studies outlining a core curriculum, framework, or skill set for AI education in healthcare.	Studies describing AI applications for education (e.g., using AI as a teaching tool).
Papers describing competencies, roles, or frameworks needed to use AI effectively to enhance care quality.	Papers measuring AI literacy maturity levels or awareness among participants without defining competencies.

Selection Process

The search yielded approximately 68 records, of which 20 publications met the inclusion criteria after abstract and full-text screening. Grey literature, including policy documents and professional guidelines, was reviewed to complement academic sources. All sources were coded for key themes such as definitional elements of AI literacy, competency domains, and educational or organizational enablers.

A.2 Expert Consultation

Expert validation was conducted through two complementary activities: (1) a roundtable discussion at Ghent University and (2) integration of findings from the VAIA (Flemish AI Academy) Learning Needs on AI in Healthcare study.

A.2.1 Ghent University Roundtable

A structured expert roundtable took place at the end of January 2025 at Ghent University's Faculty of Medicine. The roundtable was organised by the Faculty of Medicine and Health Sciences of Ghent University, VAIA and HINT.GENT (Health Intelligence Network.Gent). Approximately 40 participants—including clinicians, medical professors, and educational coordinators—attended. The session aimed to identify and validate competencies deemed essential for clinicians and medical students when interacting with AI systems. Participants were divided into two facilitated groups and asked to confirm relevance, identify missing elements, and refine the proposed competencies. Key themes included data interpretation, ethical and legal awareness, understanding AI limitations, and interdisciplinary collaboration. Although initially focused on curricular redesign at Ghent University, its outcomes directly informed refinement of the M-SHALF framework.

A.2.2 VAIA Learning Needs Study

Complementary insights were drawn from the VAIA (Flemish AI Academy) report 'Learning Needs on AI in Healthcare' (2023). The study surveyed healthcare professionals across Flanders to identify AI-related learning priorities. Findings indicated that professionals primarily require enhanced basic knowledge of AI and framework knowledge encompassing ethical, legal, and management dimensions. These findings corroborated the Ghent roundtable insights and informed the contextual layers of M-SHALF.

A.3 Integration into Framework Development

Insights from the literature review and expert consultations were integrated through a three-stage synthesis process: (1) concept extraction from the literature, (2) expert validation through Ghent and VAIA consultations, and (3) final structuring of competencies into M-SHALF's four indicator domains—awareness, application, reflection, and governance. This process ensured empirical grounding, contextual validation, and adaptability across healthcare systems.

A.4 Limitations

Although not a full systematic review, this integrative approach followed principles of transparency, reproducibility, and expert triangulation. The focus on English-language literature and European experts may limit generalizability, but inclusion of governance- and ethics-oriented domains supports broader adaptability.

Supplementary Information B. Practical Implementation Roadmap

B.1 Purpose and Scope

This appendix provides preliminary, practice-oriented guidance on how healthcare organisations of different sizes and resource levels might operationalise the Modular & Stratified Healthcare AI Literacy Framework (M-SHALF) in accordance with Article 4 of the EU AI Act. It outlines provisional implementation pathways, resource considerations, and measurable indicators intended to illustrate how the conceptual framework could be translated into sustained institutional practice, pending further validation.

B.2 Stratified Pathways by Organizational Type

Organisation type	Focus of initial implementation	Illustrative activities	Indicative timeframe
Small healthcare facilities (community clinics, general practices)	Build foundational awareness and minimal governance structure	Awareness sessions on AI basics; identification of AI tools in use; inclusion of AI-use statements in local Standard Operating Procedures;	6 – 9 months
Medium-sized hospitals or networks	Institutionalize literacy within quality and risk-management routines	Departmental literacy leads; integration into continuing-education platforms;	12 – 18 months
Large teaching or multi-site hospitals	Embed literacy within enterprise governance and compliance dashboards	Creation of AI-oversight committee; cross-functional training plans; automated tracking of literacy indicators linked to CE-mark and post-market monitoring	18 – 30 months

Timelines are indicative and assume incremental progression across the four M-SHALF domains: awareness → application → reflection → governance.

B.3 Integration into Organisational Systems

Effective implementation of M-SHALF depends on embedding literacy practices into the organisation's existing operational, governance, and human-resource systems rather than creating parallel structures. We see the integration occurring through four main channels:

1. **Training ecosystems:** M-SHALF modules can be incorporated into e-learning or professional-development platforms already used for GDPR, clinical safety, or quality management. This ensures literacy training is not a standalone activity but part of the institution's core compliance culture.
2. **Governance routines:** Literacy indicators should be linked to annual quality-management reviews, internal audits, and risk assessments, creating verifiable evidence aligned with AI Act Articles 8–15. This would anchor AI literacy pathways in regulatory and accreditation workflows.
3. **Procurement and oversight:** Include AI-use literacy expectations in internal procurement templates and informational factsheet to ensure that staff engaging with AI tools understand their regulatory status, functioning and limitations.
4. **Human-resource policies:** In a broad way, relevant M-SHALF indicators should be referenced in job descriptions, onboarding materials, and annual performance reviews. This formalises accountability and signals that AI literacy is a recognised professional competency across roles.

B.4 Resource and Feasibility Considerations

Because hospitals vary widely in size, staffing, and infrastructure, M-SHALF is designed to be proportionate, enabling organisations to build internal AI literacy within their existing capacities rather than creating entirely new structures. Implementation can be adjusted according to available human, financial, and technical resources. Here we exemplify two practical strategies illustrate how hospitals can align with M-SHALF framework without incurring significant additional costs.

From a human-resource perspective, hospitals may designate an AI Literacy Lead or Coordinator to oversee the process. This individual need not represent a new full-time role; instead, the responsibility can often be integrated into existing positions within compliance, digital transformation, or professional education teams.

In terms of financial feasibility, early-stage implementation can draw on freely available or publicly funded materials, such as the TEF-Health initiative[1], which provide foundational content on AI ethics, governance, and clinical integration. Leveraging these resources reduces costs while embedding AI literacy into familiar educational workflows. Over time, hospitals may choose to customise or expand training modules to reflect local priorities, regulatory changes, or emerging technologies.

---

[1] TEF-Health (Trustworthy, Explainable and Fair AI Systems for Health). European Commission project supporting testing and validation of AI in healthcare. Available at: https://tefhealth.eu/home#:~:text=Formally%20this%20collaboration%20is%20supported,5%20years%20of%20initial%20funding.

B.5 Indicators and Evidence Sources

Indicators provide measurable evidence that hospital staff are acquiring and maintaining AI literacy appropriate to their roles.

M-SHALF Domain	Internal Operational Indicator	Typical Evidence Source
**Awareness**	Percentage of staff completing basic AI-literacy modules; inclusion of AI-use statements in local SOPs	LMS reports; SOP repository
**Application**	Staff able to interpret CE-mark and risk classification for AI tools used in their departments; traceable documentation of human-in-the-loop decisions	Training logs; internal audit reports
**Reflection**	Frequency of post-deployment review meetings and cross-disciplinary debriefs on AI performance or incidents	Meeting minutes; risk-review records
**Governance**	Existence of an internal AI-oversight or ethics committee; inclusion of literacy metrics in quality dashboards	Governance reports; management review summaries

These indicators operationalize M-SHALF's educational objectives while producing tangible evidence for internal and external conformity assessments.

B.6 Illustrative Case Scenario

To illustrate how M-SHALF can be embedded into everyday hospital operations, we envision its application in a large tertiary hospital introducing AI-supported radiology reporting. This example demonstrates how AI literacy can be cultivated as part of routine governance and quality-assurance activities, without establishing new external structures.

Importantly, M-SHALF is both modular and stratified: hospitals can organise learning packages flexibly—sequencing, combining, or adapting modules according to their institutional priorities and resources—while ensuring that each professional group receives training aligned with its specific roles and interactions with AI systems.

Implementation unfolds through a series of interrelated steps:

- Baseline mapping: The hospital begins by identifying all AI applications in use, the departments involved, and the categories of staff who interact with these systems (e.g., clinicians, IT personnel, administrators). This mapping establishes the scope of literacy needs and highlights areas requiring tailored training.

- Role-specific literacy training: Educational content is differentiated according to staff function and level of responsibility. Radiologists and clinical users receive advanced modules on model interpretation, bias detection, and human–AI decision-making; IT staff focus on data governance, interoperability, and system monitoring; administrative and governance leaders are trained on oversight duties, documentation requirements, and risk-management obligations.

- Integration into procedures: AI-use statements are incorporated into departmental Standard Operating Procedures (SOPs), and CE-mark status is verified as part of routine equipment audits. Embedding these practices within existing operational structures ensures compliance and sustainability.

- Governance loop: The hospital's digital-governance committee monitors completion rates, literacy indicators, and feedback, reporting quarterly to senior management. This ensures institutional oversight and continuous visibility of progress.

- Continuous documentation and feedback: Findings from incident reviews, internal audits, and user feedback are used to update SOPs and inform future training cycles, closing the loop between learning and quality improvement.

Supplementary Figure 1

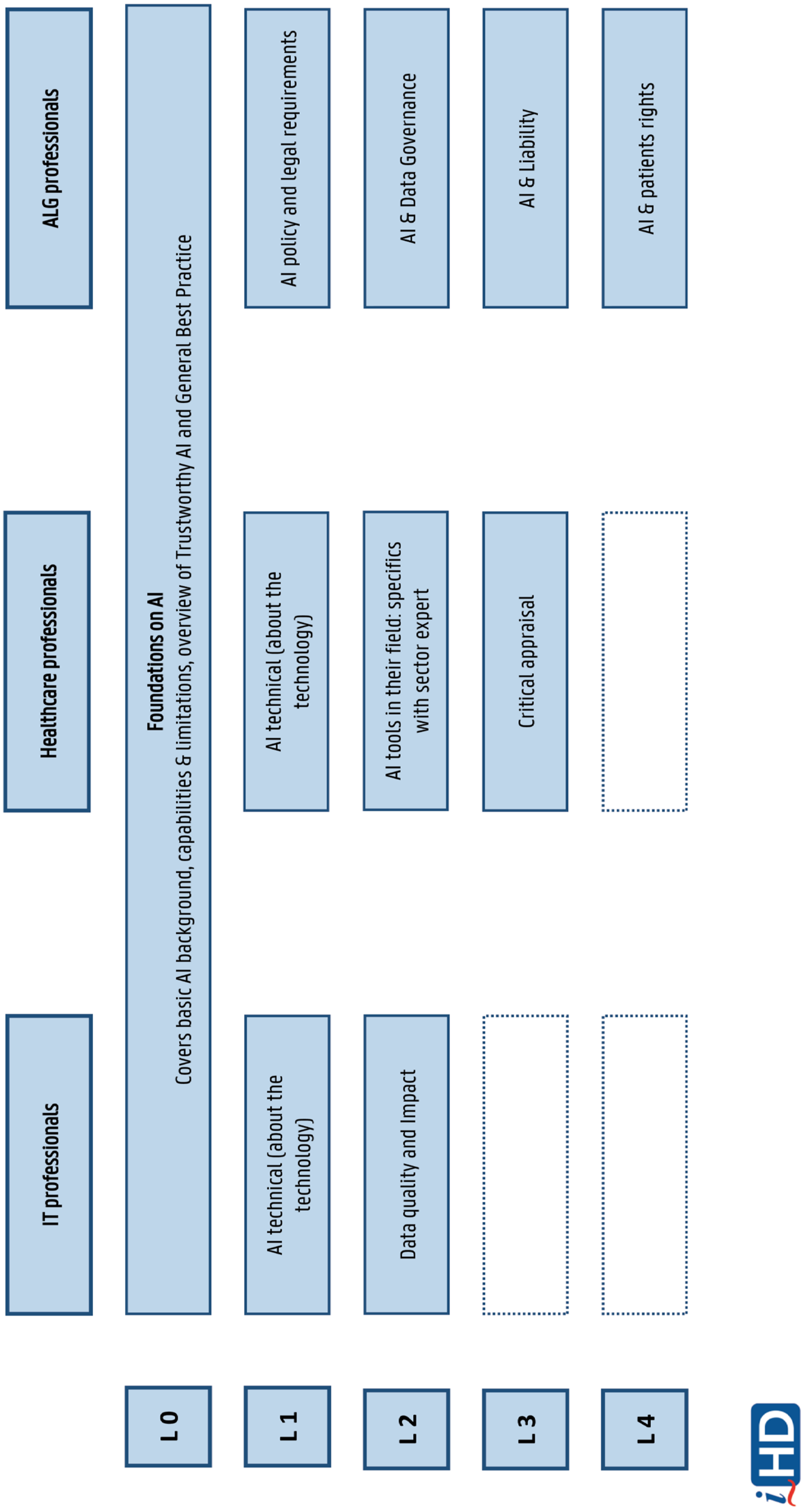

Supplementary Figure 1: *The Modular & Stratified Healthcare AI Literacy Framework (M-SHALF)*
